# A Prospective Validation Study of a Rainbow Model of Integrated Care Measurement Tool in Singapore

**DOI:** 10.5334/ijic.2021

**Published:** 2016-04-08

**Authors:** Milawaty Nurjono, Pim P. Valentijn, Mary Ann C. Bautista, Lim Yee Wei, Hubertus Johannes Maria Vrijhoef

**Affiliations:** Saw Swee Hock School of Public Health, National University of Singapore, Singapore; Scientific Centre for Care and Welfare (Tranzo), Tilburg University, Tilburg, The Netherlands; Jan van Es Institute, Netherlands Expert Centre Integrated Primary Care, Almere, The Netherlands; Saw Swee Hock School of Public Health, National University of Singapore; National University Health System, Singapore; Department of Patient and Care, University Hospital Maastricht, Maastricht, The Netherlands; Vrije Universiteit Brussels, Brussels, Belgium

**Keywords:** integrated care, measurement, validation

## Abstract

**Introduction:**

The conceptual ambiguity of the integrated care concept precludes a full understanding of what constitutes a well-integrated health system, posing a significant challenge in measuring the level of integrated care. Most available measures have been developed from a disease-specific perspective and only measure certain aspects of integrated care. Based on the Rainbow Model of Integrated Care, which provides a detailed description of the complex concept of integrated care, a measurement tool has been developed to assess integrated care within a care system as a whole gathered from healthcare providers’ and managerial perspectives. This paper describes the methodology of a study seeking to validate the Rainbow Model of Integrated Care measurement tool within and across the Singapore Regional Health System. The Singapore Regional Health System is a recent national strategy developed to provide a better-integrated health system to deliver seamless and person-focused care to patients through a network of providers within a specified geographical region.

**Methods:**

The validation process includes the assessment of the content of the measure and its psychometric properties.

**Conclusion:**

If the measure is deemed to be valid, the study will provide the first opportunity to measure integrated care within Singapore Regional Health System with the results allowing insights in making recommendations for improving the Regional Health System and supporting international comparison.

## Publisher's Note

The PDF version of this article was originally published using a different layout. It has now been re-laid-out in a format consistent with the rest of the volume.

## Introduction

The increasing demand for health services, the fragmentation of health systems, the changing health needs and the ever-increasing influence of economic, political and social factors on health and healthcare delivery, have collectively contributed to the shift in focus of health systems [1]. Against such a complex landscape, the evolution of the healthcare system from the traditional disease-centric model to a person-focused healthcare system [2] has become imperative. Person-focused care takes into account all the needs of a person over time. It seeks to provide appropriate care to cope with a person’s holistic needs in a more appropriate and timely way and so to deliver better quality care [3]. Integrated care supports person-focused care through coordinating services around a person’s needs to improve accessibility, affordability and quality of healthcare, especially for those who suffer from multiple chronic illnesses [4].

Like many other developed nations, Singapore’s population is rapidly aging with an increasing prevalence of chronic and complex illnesses. It has been estimated that Singapore citizens over 65 years of age will triple to 900,000 by 2030 [5]. As of 2002, 83% of all registered deaths in Singapore were due to chronic illnesses. These rapid demographic changes inevitably exert a significant demand on the health system and raises overall healthcare costs, rendering the current acute-centric provision of care unsustainable. In response to such challenges, there is a realisation for the need of an integrated healthcare system that links the primary, secondary and tertiary care settings to provide care across different stages of the care continuum. As such, the Ministry of Health initiated a strategy to reform the existing health system into a more inclusive and integrated one [6]. The health system was re-categorised into six Regional Health System by geographic location. Every Regional Health System is anchored by a Public Healthcare Cluster, which oversees one or more public acute hospitals, and is also responsible for the integration of healthcare services within their region through strengthening primary care, developing family medicine clinics and empowering the local communities. The intermediate long-term care system was also strengthened through manpower and infrastructure funding and a better financial subsidy framework. The Regional Health System comprises a network of health care (e.g. General Practitioners, Community Hospitals and nursing homes) and social care providers (e.g. Senior Activity Centres, Grassroots, etc.), potentially harnessing a multidisciplinary care team approach that involves the acute, step-down, primary, mental health and community care sectors to provide integrated care for patients throughout their healthcare journey [7].

However, despite the growing needs to provide integrated care, there is no common definition for integrated care thus far, and existing definitions remain debatable due to its conceptual ambiguity [8]. The diversity in definitions of integrated care has been based on the perspectives of various actors in the integration efforts. Integrated care denotes different things for different people and has been used interchangeably with managed care in the United States of America, shared care in the United Kingdom, transmural care in the Netherlands and comprehensive care or disease management in other parts of the world [9]. The majority of existing definitions of integrated care were developed from a narrow, disease-oriented perspective [9] that typically focuses on the integration of organisations and professional activities without much regard for the bio-psychosocial aspects, which takes into consideration all relevant determinants of health including biological, psychological and social factors [10] and patients’ perspectives on the quality of healthcare delivery [11]. The narrow focus limits the generalisability and applicability of these definitions to broader settings.

It is essential to recognise that since health problems are not contributed solely by biological factors, broad inter-sector approaches which also tackle the psychological and social determinants of health are needed to meet the health needs of the population especially those living with chronic conditions. The close link between social and medical issues highlights the increasing influence of the non-medical determinants of individual and population health, suggesting the importance of extending healthcare provision to the community. Hence, integration across various levels of medical care and public health—including support for social services such as education and housing—should be fostered within the community. The population-based approach supports the provision of a continuum of primary, secondary and tertiary medical care within the community, to overcome the challenges faced by many healthcare systems [12,13]. In addition, to contain the ever-increasing cost of healthcare, it is crucial to go beyond the curative model of care and include preventive efforts that can reduce morbidity and mortality within specific populations, potentially reducing subsequent health care utilisation, and thus costs.

Given the conceptual ambiguity of the integrated care concept, it is not fully understood what constitutes a well-integrated health system, posing a significant challenge in measuring the level of integrated care. Most existing measurements of integrated care are limited in scope, focusing on measuring clinical, vertical or horizontal integration separately and from the perspective of either health and social care providers or patients [14]. In addition, existing measures only measure aspects of integrated care that are measurable and considered important from the developers’ perspective, which limits a more comprehensive measurement of integrated care [14]. This is primarily so because the broad system definition of integrated care has failed to bring practical relevance to practitioners and policy makers, forcing them to adopt the narrowly defined concept. This key limitation points towards the need for a broad, yet consistent, and comprehensive conceptual framework that can be applied to any integrated care setting. Such a framework can facilitate communication, policy formulation, program development and evaluation of integrated care within and between settings [15].

## The Rainbow Model of Integrated Care

**Figure 1:**
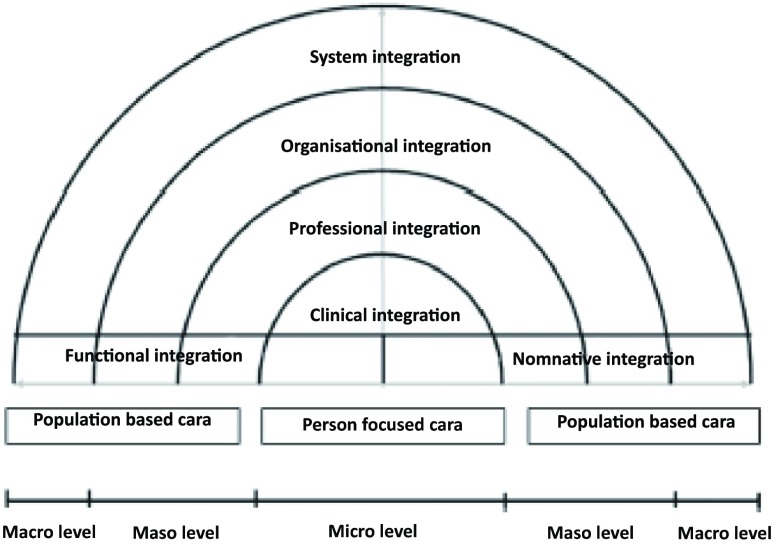
The rainbow model of integrated care based on integrative functions of primary care [19].

Developed through literature reviews [16] and validated by a series of Delphi panels in the Netherlands, [17] and expert panels in international conferences held in Singapore and Brussels [18], the Rainbow Model of Integrated Care provides a detailed description of integrated care from the primary care perspective [19]. As depicted in Figure 1, the Rainbow Model of Integrated Care provides conceptual clarity by combining the concept of primary care and integrated care and is considered useful for understanding the complexity of integrated care [20]. It describes dimensions that play inter-connected roles on the macro- (system integration), meso- (organisational and professional integration) and micro-level (clinical, service and personal integration) and dimensions (functional and normative integration) that enable the integration between different levels within a health system in the provision of continuous, comprehensive and coordinated delivery of services to the individual and population [19]. The Rainbow Model of Integrated Care provides a comprehensive definition of integrated care that considers both the person-and population-based focus.

The Rainbow Model of Integrated Care highlights the importance of considering biopsychosocial factors in the provision of patient- and population-centred integrated care throughout the entire population. The person-focused and population-based perspectives, as proposed by Rainbow Model of Integrated Care, can potentially bridge the gap between health and the social system. Although the Rainbow Model of Integrated Care was developed from the primary care perspective, the dimensions identified are relevant and can be contextualised to any integrated care setting, particularly given the shared fundamental focus of both primary care and integrated care in bringing the healthcare system and other human service systems together in the pursuit for improved population health outcomes [21]. The first comprehensive measure has since been developed to measure the level of care integration based on the Rainbow Model of Integrated Care (not as a formal reference). This measure aims to capture the prevailing opinions and perspectives of all actors involved in the care integration processes in settings where integration is planned to be implemented, or is already being implemented. However, since its development, the integrated care measure has only been administered in the Netherlands (data is being collected). To use the measure in other countries, it is important to examine its relevance and statistical validity in an appropriate integrated care setting elsewhere. This study therefore aims to validate the Rainbow Model of Integrated Care integrated care measure in the Singapore Regional Health System. Examining the validity of this tool within the Singapore Regional Health System will provide an opportunity for further validation of the measure which to date has only been used in the Dutch context.

## Methods

Our study adopted the Rainbow Model of Integrated Care-based integrated care measure developed by Valentijn et al. [19] that measures the level of integrated care as a whole—from a broad, systems oriented point of view from healthcare providers’ and managerial perspectives. The original measure that can be made available upon request from the authors consists of 44 items grouped into eight dimensions corresponding to eight domains of integrated care. The first two dimensions cover the scope of integrated care and include statements related to the person-focused (5 items) and population-based (4 items) approaches within the integrated care setting. The next four dimensions cover the type of integrated care and include statements related to the domains of clinical integration—the coordination of care process for individuals between different professionals (5 items), professional integration—shared accountability between different professionals within the partnership (6 items), organisational integration—the collaborative governance mechanisms between different organisations (6 items) and the system integration—the linkages with external environment in which the care integration is built (5 items). The last two dimensions correspond to the category of enablers, which complete the six original domains in the Rainbow Model of Integrated Care framework. They include statements that relate to the domains of functional which describes the essential support functions and activities (7 items) and normative integration that is related to essential social and cultural factors within the partnership (6 items). The measure uses 5-point Likert scale ranging from never to all the time. An additional option (not sure/don’t know) which allows participants to indicate if he or she is not sufficiently aware of the activities specified to provide an answer is also included on the measure. Higher scores on the Rainbow Model of Integrated Care are expected to represent higher level of integrated care.

The validation of the integrated care measure within the Regional Health System will be conducted in two consecutive phases.

### Phase 1: face and content validity

In Phase 1, three representatives from each Regional Health System planning offices including senior managers and Regional Health System senior office staff will be invited to review the outlook and content of the integrated care measure. Through a face-to-face interview, each participant will be asked to review each item on the survey, comment on their wordings, interpret meaning, give an opinion about the relevance of the items in the Singapore context, identify redundant items to be removed and suggest new items to be added to the measure. The participants will also be asked to comment on the length of the survey and time taken to complete it.

Based on the collective opinions gathered from the individual experts and iterative consultations among the research team and the measure’s developer, adaptation to the original Rainbow Model of Integrated Care measure will be made. Wordings will be refined, and additional items will be added as deemed necessary by the expert reviewers. Before the actual administration to the target participants to test the tool’s psychometric properties, the adapted Rainbow Model of Integrated Care measure will be tested with the initial reviewers from the Regional Health System and other independent healthcare researchers for further comments.

### Phase 2: assessment of psychometric properties of the modified integrated care measure

Approximately 200 healthcare providers from two Regional Health Systems (the National Healthcare Group and the National University Health System) will be invited to complete the adapted integrated care measure online (with the assistance of the Regional Health System planning office). The participants will include senior managers, Regional Health System office staff, and healthcare professionals (doctors, nurses, physiotherapists and pharmacist and case managers). These participants must be involved in the planning and development of the Regional Health System and have a good understanding of the processes of integrated care within the Regional Health System. As healthcare providers may be involved in multiple roles within the healthcare system, brief explanatory notes about integrated care and the Regional Health System will be included preceding the integrated care survey. This is to clarify the context in which the participants are providing opinions.

Based on the responses gathered analysis will be conducted by using the statistical software package, Predictive Analytics Software / SPSS version 18. Distribution of the variables will be first examined to assess the response variability and missing data. Participants with more than 30% missing data on a scale were excluded from the analysis. Cronbach’s alpha will be calculated to determine the internal consistency of the survey. The survey will be considered to be reliable for use within the sample population if the Cronbach’s alpha ranges between 0.70 and 0.95 [22]. The inter-item correlation will then be assessed to determine the uni-dimensionality of the survey measure. After which, Bartlett’s test of sphericity and the Keiser-Meyer-Olkin measure of sampling adequacy will be tested to determine if factor analysis can be conducted to establish the construct validity of the measure. Only when Bartlett’s test is significant and Keiser-Meyer-Olkin value falls near 1, factor analysis will be conducted. Exploratory Factor analysis using the principal component analysis will be performed to examine the best fit for the data and identify the reliable and redundant items to be retained and deleted respectively based on the eigenvalues, scree plot, and the total variance explained. Furthermore, deleted-item reliability correlations and inter-correlations between dimensions will also be calculated to establish construct validity. A moderate correlation of ≥0.70 is expected if the constructs included in the survey are valid [22]. Finally, the convergence between the scores calculated from the modified integrated care survey and qualitative responses of the Regional Health System representatives collected as part of the online survey will also be assessed.

## Potential implications

The lack of a universal definition and empirical framework to comprehensively describe integrated care hampers the communication, policy formulation, programme development and evaluation of integrated care. To date, there is a paucity of standardised measures to comprehensively measure integrated care. This study presents an opportunity to test a measurement tool that was developed from a systematic review of international literature and a comprehensive empirical framework of integrated care, which has been tested rigorously through three Delphi expert panel discussions in the field of integrated care. The inclusion of relevant stakeholders involved in the integration processes in the Singapore context and the rigorous validation methodology adopted by this study will provide reliable information to determine the validity and accuracy of the survey in a different setting outside of where it was first developed. This study will also suggest the survey’s suitability for use in Singapore, where the level of integrated care has never been evaluated.

If the integrated care measure is deemed reliable and valid for use in Singapore, the measure can potentially be used as an outcome measure to monitor the progress of the Regional Health System as the domains measured on the integrated care survey are aligned with the objectives of the Regional Health System. Using a validated measure will also allow accurate and reliable assessment of the baseline and subsequent performance in integrating care within and across the Regional Health System, identifying areas per region or for all the Regional Health System to be strengthened based on respective stakeholders’ perspectives. Determinants of success in respective regions can also be shared among the regions so that each Regional Health System can learn from each other.

### Strengths and limitations

The strengths of this study potentially lie on the nature of the measurement tool used and the reliability the study methodology adopted. The integrated care measure adopted in this study was rigorously developed and comprehensively assesses various dimensions of integrated care from the healthcare providers’ perspectives, thus allowing an overall evaluation of the healthcare system to be made. Furthermore, as the measurement tool has also been used to measure the level of integrated care in other countries, using the same tool in Singapore will allow for international comparisons.

However, although a modified version of the survey is expected to be suitable for use in Singapore, it must be recognised that the original was based on an assumption that integrated care could be grown from the primary care level. Given the current weakness in Singapore’s first level of care, the measurement tool may not be entirely applicable for Regional Health Systems in Singapore since the Regional Health Systems are organised in geographical units around key acute hospitals within each respective region. In addition, as the measure only captures the perspectives of healthcare providers, it is also important to consider including social care providers and patients’ perspectives to give a more complete and accurate measurement of integrated care within the Regional Health System. Learning from healthcare users will also help healthcare providers understand the health needs and cater to them accordingly. This perspective is looked at in another study currently being undertaken at the National University Health System.

## Conclusion

This study describes the methodology of the validation process of the Rainbow Model of Integrated Care-based measurement tool in the Singapore Regional Health System context. The validation process includes the assessment of the content of the measure and its psychometric properties. If the integrated care measure is deemed reliable and valid for use in Singapore, this study potentially provides the first opportunity to measure integrated care within and between the Regional Health System, with the results allowing insights in making recommendations for improving the Regional Health System and supporting international comparison.

## Competing Interests

The authors declare that they have no competing interests.
